# Developmental trends in early childhood and their predictors from an Indian birth cohort

**DOI:** 10.1186/s12889-021-11147-3

**Published:** 2021-06-06

**Authors:** Beena Koshy, Manikandan Srinivasan, Anuradha Bose, Sushil John, Venkata Raghava Mohan, Reeba Roshan, Karthikeyan Ramanujam, Gagandeep Kang

**Affiliations:** 1 Developmental Paediatrics Unit, Christian Medical College, Vellore, 632004 India; 2 Wellcome research Unit, Christian Medical College, Vellore, 632004 India; 3 Community Health, Christian Medical College, Vellore, 632004 India; 4 Low Cost Effective Care Unit, Christian Medical College, Vellore, 632004 India

**Keywords:** Early childhood, Developmental trends, Socio-economic position, Home environment, Maternal factors

## Abstract

**Background:**

Early childhood developmental pattern analyses not only project future cognition potential, but also identify potential risks for possible intervention. The current study evaluates developmental trends in the first 3 years of life and their predictors in a low and middle income country setting.

**Methods:**

Trends of early childhood development at 6, 15, 24 and 36 months of age and their predictors were explored in a longitudinal community-based birth cohort study in an urban slum in Vellore, South India. Development was assessed using the Bayley Scales of Infant and Toddler Development-III (BSID-III).

**Results:**

The birth cohort enrolled 251 children with 94, 91, 91 and 87% follow-up at 6, 15, 24 and 36 months respectively. Child development domains of cognition, language, motor and social skills showed a significant decline in scores between 6 and 36 months of age. Higher socioeconomic position (SEP) and nurturing home environment contributed to increase in cognition scores by 1.9 and 0.9 units respectively. However, stunting caused a decline in cognition scores by 1.7 units. Higher maternal cognition, higher SEP, and caregiver responsivity positively contributed to language change over time, while higher maternal depression contributed negatively. An enriching home environment, growth parameters and blood iron status had positive association with change in motor skills.

**Conclusions:**

A triple intervention plan to enhance home environment and nurturance, early childhood nutrient supplementation, and maternal education and well-being might prevent child developmental decline in high risk settings.

**Supplementary Information:**

The online version contains supplementary material available at 10.1186/s12889-021-11147-3.

## Background

It is estimated that around 250 million young children living in the low and middle income countries (LMIC) risk poor development and sub-optimal livelihood potential [1, 2] because of exposure to twin risks of extreme poverty and stunting [3]. Describing developmental trajectory patterns in the LMIC not only helps in understanding developmental variations different from conventional standards, but also aids in identifying sensitive periods and specific risk factors including adverse childhood experiences. Region-specific analysis of developmental trajectory patterns can in addition help to custom-make specific developmental intervention, which can be later incorporated into public health policy.

Sensitive periods for optimal childhood development include the time period from conception to 2 years of age considered the first-1000 days of life [4] and from two to 5 years of age, the second-1000 days of life [5, 6]. Large multi-national birth cohort studies have shown complex interactions among early childhood infections, nutritional intake, family socio-economic position and home environment, affecting cognition at 2 years [7] as well as 5 years of age [8]. Risk factors including foetal growth restriction manifesting as low birth weight [9], absolute poverty [2], early childhood stunting [2, 5], micronutrient deficiency [10], poor sanitation [11], diarrhoea [12, 13], maternal depression [14] and sub-optimal home environment [15, 16] can adversely impact early child development with persisting effects on later school cognition, learning and behaviour [1, 2, 5].

Early childhood developmental trajectory analyses have been predominantly restricted to cognition [9, 17, 18], with a few evaluating other developmental domains such as social skills [19]. Group based trajectory modelling of a large Chinese birth cohort showed four different patterns of cognition paths in the first 24 months of life [9], while longitudinal social profiles of Japanese children between two and 5 years of age showed three different trajectories [19]. Development of neuropsychological functions in Indian children was described as “non-linear, heterogeneous and protracted” in a cross-sectional study [20]. Indian birth cohort follow-up studies, as part of large multinational projects, have predominantly evaluated early childhood infection/growth in relation to later age cognition [12, 21, 22] barring a few evaluating early childhood growth-development linkage [23]. There have been few studies exploring evolution of all domains of development over time in the early years, especially in a LMIC setting. The current study was planned to evaluate the trends of child developmental domains of cognition, language, social and motor skills in the first 3 years of life and analyse their predictors. It is hypothesized that socio-economic position, home environment, maternal cognition and micronutrient status will influence developmental domains.

## Methods

### Settings and subjects

The present study was done as an independent sub-analysis of a large prospective, multinational longitudinal birth cohort study conducted across eight different nations across the world -‘The Etiology, Risk Factors and Interactions of Enteric Infections and Malnutrition and the Consequences for Child Health and Development (MAL-ED) Network’ [24]. The Indian study site was a densely populated urban slum in Vellore, South India and covered a population of 12,000 [25]. Children were enrolled at birth by consecutive sampling and details of enrollment, exclusion criteria, follow up and details are available in previously published articles from the same birth cohort [26–28]. The original birth cohort enrollment and consequent follow-ups were approved by the Institutional Review Board of Christian Medical College, Vellore and children were recruited at each stage after informed parental consent.

### Measures

#### Bayley scales of infant and toddler development-III (BSID-III)

The Bayley Scales of Infant and Toddler Development-III (BSID-III) assesses development in the domains of motor, language, cognition and social skills between 1 and 42 months of age [29], and details of administration are available with proposed methodology [30], as well as community level conduct already published [27, 28]. BSID-III was administered at 6, 15, 24 and 36 months of age. Individual domain quotient was calculated from the raw score using child’s chronological age.

#### The WAMI measure for socio-economic position

The WAMI measure with components of access to improved water and sanitation, assets, maternal education and total household income is a simplified measure of socio-economic position (SEP) developed during the MAL-ED study, [31] and details are provided in other published articles from the same cohort [26–28].

#### The HOME scale

The Home Observation for the Measurement of the Environment (HOME) scale (Infant/Toddler version) analyses the home environment of the child including stimulation and support and is considered the gold standard with good psychometrics [32, 33]. Both physical and interactive home environment are observed and scored in this measure, specifically mother-child interactions including responsiveness.

The modified version has six subscales consisting of total 48 items with subscales of appropriate play materials, avoidance of restriction and punishment, organization of the environment, parental involvement, responsiveness to parent, and variety in daily stimulation [34]. The HOME scale was administered by a trained social worker, who observed the home environment for 45–60 min at 6, 24 and 36 months of child’s age. Supplementary information was obtained using a mother/caregiver interview as per the MAL-ED study protocol [35]. Further details about this measure is provided in another published article from the same birth cohort [26].

Maternal cognition was assessed by Raven’s progressive matrices, a scale of non-verbal reasoning at 6–8 months of child’s age [36]. The Self Reporting Questionnaire-20 (SRQ-20), developed by the World Health Organisation to assess depressive symptoms in low-resource settings, was used to assess maternal psychological disturbances at 1,6, 15, 24 and 36 months of child age [37]. The total SRQ score was calculated using 16 items, which yielded one factor structure [38].

### Blood collection

Blood samples were collected at 7, 15, 24, 36 and 60 months; and details of collection, testing mechanisms and methods are published [28]. Samples were tested for hemoglobin (7, 15, 24, and 36 months); ferritin assays (at 7, 15 and 24 months) and blood lead levels (at 15, 24 and 36 months). As inflammation can influence serum ferritin levels, both transferrin (R) and ferritin (F) levels were utilized to find total body iron levels using the formula, where positive values indicated iron reserves [39]:

$$ \mathrm{Body}\ \mathrm{Iron}\ \left(\mathrm{mg}/\mathrm{kg}\right)=\left(\frac{-\left(\log \left(\frac{R}{F} ratio\right)-2.8229\right)}{0.1207}\right) $$

### Data entry

Data entry was made in the double entry database system managed by Data Coordinating Centre (DCC) of the MAL-ED study. Data collection forms filled by the field workers were authenticated by the field supervisor before entering into the database [24].

### Statistical analysis

The outcome variable, BSID scores, assessed at 6, 15, 24 and 36 months of age was summarized using mean and standard deviation (SD) under specific domains namely - cognition, language, motor and socio-emotional domain. Independent predictors such as maternal depression score, maternal cognition and various domains of HOME inventory scale were summarized as mean and SD. Height-for-age and weight-for age scores below − 2 SD were categorized as stunted and underweight respectively. Values of blood iron measured at 7, 15 and 24 months were considered for analysis and the missing values were replaced with the average of measurements available at the other time points. Blood lead levels measured at 15, 24 and 36 months were averaged to obtain mean lead level. Domain-wise score of cognitive development was compared across the time points using repeated measures ANOVA test. Factors predicting cognitive development scores across the four time points were measured using the generalized estimating equations (GEE) – population averaged model based on the following equation,

Cognition development_*ij*_ = α_i +_ β_1_ Gender + β_2_ Stunted_*ij*_ + β_3_ Underweight_*ij*_ + β_4_ Mother’s cognition scores+ β_5_ SEP_*ij*_ + β_6_ Body iron_*ij*_ + β_7_ Mean blood lead + β_8_ Mother’s depression scores_*ij*_ + β_9–14_ Domains of HOME Inventory scale + ε_*ij*_*.*

where *‘i’* and *‘j’* refers to children and time points of measurements respectively, and, ε_ij_ representing the random error. Multicollinearity of the covariates included in the model was tested using variance inflation factor and none of the variables were observed to be collinear. GEE model was used to adjust for clustering at the subject level, because of repeated measurements on children. Subject ID of study children was used as the clustering variable in the model. We specified exchangeable correlation structure in the model, based on the quasi Information Criteria (QIC) and robust standard errors were estimated [40]. Beta co-efficients along with 95% confidence interval have been reported for the independent predictors. Model fit was assessed using Wald statistics and p less than 0.05 was considered as statistical significance. Data analysis was done using Stata version 13 (StataCorp. 2013. Stata Statistical Software. Release 13. College station, TX: StataCorp LP).

## Results

In the original birth-cohort, 251 newborns were enrolled after registering 301 pregnant mothers. 50 children were excluded as per the exclusion criteria: another sibling registered in the MAL-ED study (*n* = 8), medical comorbidities in children (*n* = 7), multiple pregnancy (*n* = 1), pre-existing plan to migrate (*n* = 5), mother not available for consent (*n* = 9), combination of two or more of the above mentioned reasons (*n* = 10) and mothers/parents refused participation (*n* = 10). Subsequent follow ups at 6, 15, 24 and 36 months had 235, 229, 228 and 218 children respectively. Migration was the main cause for the loss to follow-up, as illustrated in other published articles from the same cohort [28].

The Vellore birth-cohort had a mean birth weight (SD) of 2.89 (0.44) kg and 17% of newborns weighed less than 2.5 kg. More than 80% of families had monthly income less than 5000 Indian rupees (70 USD). The cohort had girl predominance at birth (56%) as well as subsequent follow-ups. Cohort characteristics at 6 and 36 months are summarized in Table 1, with details of 6, 15, 24 and 36 months in Supplementary Table 1.

**Table 1:** Baseline characteristics of the birth cohort established in Vellore in 2010

Variables	6 months (*n* = 235)	36 months (*n* = 218)
Sex (%)	Female: 129 (54.89%)	116 (53.21%)
HOME total score, mean (SD)	40.77 (3.24)	40.27 (3.18)
Domain-wise score of HOME inventory score, mean (SD)		
Emotional and verbal responsivity of caregiver,	10.60 (1.10)	11 (0.07)
Avoidance of restriction and punishment	4.68 (0.52)	4.12 (0.46)
Caregiver promotes child development	4.67 (0.69)	4.96 (0.20)
Organization of physical and temporal environment	10.32 (1.37)	9.49 (1.49)
Provision of appropriate play materials	2.24 (0.69)	2.66 (0.48)
Opportunities for variety in daily stimulation	5.38 (1.57)	5.75 (1.56)
Cleanliness of child	2.87 (0.43)	2.28 (0.85)
WAMI score, mean (SD)	0.453 (0.15)	0.54 (0.17)
Maternal depression (SRQ) score, mean (SD)	4.53 (3.74)	3.82 (3.53)
Maternal cognition raw score, mean (SD)	43.90 (10.49)	–

**Fig. 1:**
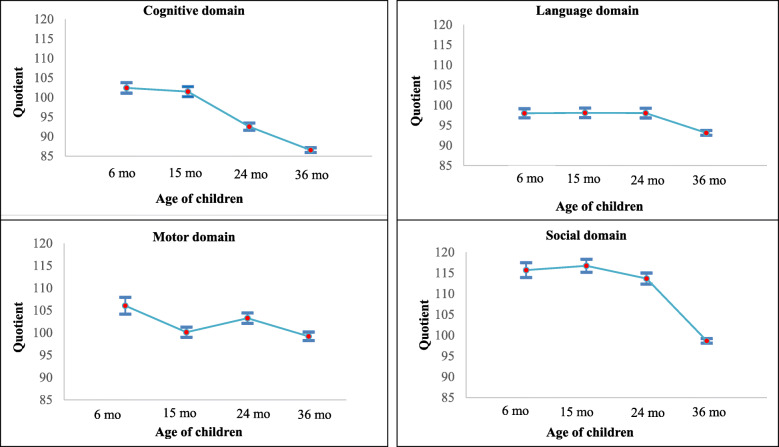
Domain wise scores obtained in Bayley scales of infant development assessed between 6 and 36 months of age in children of MAL-ED cohort, Vellore

Developmental domain quotients of cognition, language, motor and social skills significantly differed between 6 and 36 months of age (Table 2) with both cognition and social domains showing decline of more than 15 points (Fig. 1).

**Table 2:** Mean (SD) domain quotient scores of Bayley Scales of Infant Development -III represented domain-wise across 6, 15, 24 and 36 months in MAL-ED children (*N* = 216)

Domains	6 months	15 months	24 months	36 months	*P*-value
Cognition	102.43 (9.93)	101.48 (9.57)	92.52 (6.91)	86.53 (4.55)	< 0.0001
Language	98.0 (8.38)	98.10 (8.90)	98.05 (8.98)	93.13 (4.44)	< 0.0001
Motor	106.04 (14.08)	100.11 (8.51)	103.26 (8.69)	99.19 (7.19)	< 0.0001
Social	115.69 (13.4)	116.74 (11.64)	113.66 (9.98)	98.66 (4.13)	< 0.0001

Developmental quotient scores scores under each domain were compared across time points using repeated measures ANOVA test

Analysing factors responsible for the change in cognition scores between 6 and 36 months, higher SEP, body iron levels and HOME factors of parental responsivity and provision of appropriate play materials positively predicted cognition (Table 3). The maximum positive association was with SEP followed by HOME factors, while stunting had the maximum negative impact. Higher maternal cognition, higher SEP, and caregiver responsivity positively contributed to language change over time, while higher maternal depression contributed negatively. Maximum effect size on language change was seen with SEP.

**Table 3:** Factors associated with child development scores measured at 6, 15, 24 and 36 months in Vellore cohort of MAL-ED study (*N* = 216)

Factors	Adjusted beta co-efficient with 95% CI		
	Cognitive domain^e^	Language domain^e^	Motor domain^e^
Female	0.51 (−0.76–1.77)	1.14 (− 0.07–2.35)	1.54 (− 0.09–3.16)
Mother’s cognition score	0.03 (− 0.03–0.09)	**0.09 (0.03–0.15)**	**0.09 (0.01–0.17)**
Stunted (HAZ < − 2SD)^a^	**− 1.66 (− 3.18 - -0.14)**	0.13 (− 1.41–1.66)	**− 1.95 (− 3.63 - -0.26)**
Underweight (WAZ < − 2SD)^b^	−0.54 (− 1.91–0.82)	−1.34 (− 2.80–0.13)	**−2.41 (− 4.12 - -0.70)**
SEP (WAMI index)^c^			
Middle (33rd – 66th percentile)	1.08 (− 0.55–2.71)	1.39 (−0.14–2.79)	0.83 (− 0.96–2.63)
High (>66th percentile)	**1.86 (0.28–3.43)**	**1.73 (0.22–3.24)**	1.59 (− 0.35–3.52)
Body iron levels	**0.18 (0.06–0.29)**	0.05 (− 0.05–0.14)	**0.30 (0.17–0.43)**
Mean blood lead levels^d^	**−**0.03 (− 0.13–0.07)	−0.07 (− 0.18–0.04)	−0.04 (− 0.18–0.11)
Mother’s depression scores	0 (− 0.18–0.17)	**− 0.23 (− 0.41 - − 0.04)**	-0.04 (−0.25–0.17)
Domain scores of HOME Inventory scale			
Emotional and verbal responsivity of caregiver	**0.92 (0.13–1.97)**	**1.0 (0.23–1.87)**	1.30 (− 0.03–2.63**)**
Avoidance of restriction and punishment	0.68 (− 0.56–1.92)	0.21 (− 1.03–1.45)	**1.91 (0.46–3.36)**
Caregiver promotes child development	1.03 (−0.89–2.94)	1.09 (− 0.47–2.66)	0.68 (− 1.63–2.98)
Organization of physical and temporal environment	0.11 (−0.32–0.55)	0.39 (− 0.49–1.26)	− 0.12 (− 0.73–0.49)
Provision of appropriate play materials	**0.98 (0.06–1.94)**	0.39 (− 0.49–1.26)	**1.38 (0.09–2.66)**
Opportunities for variety in daily stimulation	−0.02 (− 0.46–0.41)	0.14 (− 0.12–0.83)	−0.40 (− 0.94–0.15)

^a^Children with height-for-age Z scores < − 2 SD were classified as stunted

^b^Children with weight-for-age Z scores < − 2 SD were classified as underweight

^c^WAMI index was based on water and sanitation facility, household assets, mother’s education, and total income

^d^ Mean blood lead levels were obtained by taking average of lead values measured at 15, 24 and 36 months of age

^e^Dependent variables considered for analysis were cognition, language, and motor domains of BSID-III scores. Regression co-efficients were derived using generalized estimating equations and the model fitness was assessed for each domain separately using Wald statistics and *P* value was noted as < 0.0001

Factors positively contributing to change in motor scores included higher maternal cognition, higher weight and height for age scores, higher body iron levels and positive home factors of avoidance of restriction and punishment, and availability of appropriate play materials. Both HOME factors and growth parameters had high association with change in motor skills. Body iron levels positively influenced social domain change over time with beta-coefficient (95% CI) of 0.20 (0.05–0.34), while blood lead levels had a negative influence with beta-coefficient (95% CI) of − 0.22 (− 0.34- -0.09) in univariate analyses. There were no significant associations in multivariate analysis, thus not reported in Table 3.

## Discussion

This prospective longitudinal birth cohort follow-up analysis from urban Vellore evaluated developmental trends in the domains of cognition, language, motor and social skills; and predictors for individual domain change over time. All developmental domain quotients showed a decreasing trend over time with cognition and language domains dropping more than 15 points between 6 and 36 months of age. Children in the highest tertile of SEP had better cognition and language scores over time. Stunted children had poorer cognition and motor scores over time. Home environmental factors of parental responsivity affected changes in cognition and language scores, while punishment avoidance and toy availability influenced the motor score change. Higher maternal cognition affected changes in both language and motor domains, while maternal depression adversely affected language scores over time. Mean body iron levels was associated with changes in both cognition and motor skills. There was a good level of follow-up in the current study with 94% at 6 months, 91% at 24 months and 87% at 36 months.

Child development evolves over time and developmental measures such as BSID-III have shown weak predictability of school age cognition [41], motor skills [42], and behaviour [43]. Despite this, BSID is the most common developmental tool used across the globe, as it is a sensitive measure of child development [29]. Though child developmental process and sequence are uniform across the world, there can be differences in the rate of achievement between populations. Trends of all developmental domains in an urban low-income setting as shown in this study can be useful for clinical practice in such settings, academic understanding, and analysis of risks contributing to any setback. Though the current analysis has not done a complete trajectory exploration, the birth cohort showed a decreasing trend in all developmental domains’ quotients. This is in discordance with another large rural birth cohort study reported from China, where more than 90% showed an increasing trend in cognitive development between 6 and 24 months of age [9]. The Vellore birth cohort is predominantly a low-income urban slum setting with additional environmental and nutritional challenges, which have been explored in the current analysis. Similar results were reported from another study conducted in rural India, where recruited infants showed a decline in scores in fine motor, receptive and expressive language skills and visual reception over a 6-month follow-up period [23]. A previous birth cohort follow up done in other urban slums of Ramnaickanpalayam, Chinnallapuram and Kaspa in Vellore had also revealed decline in cognition scores between three and seven years of age [44].

The SEP including household wealth affects linear growth [45, 46], development [2] and cognition [15, 47] in children. Childhood SEP is shown to influence neural development particularly of the language and executive function areas in the brain [48]. Better SEP can result in improved nutrition, better sanitation, less infection and enhanced interactive experiences, all of which can improve child development especially language as shown in the current study.

Stunting had independent associations with cognition and motor scores despite correction with SEP, HOME status and micronutrient levels in the current study. Stunting has been shown to affect development and cognition; thus integrated interventions have been suggested to improve both linear growth and child development [2, 5]. A rural study conducted in Telangana, South India found similar results where height z-scores at enrollment had a positive association with all child development domains, the effect of which was attenuated by a nurturing home environment [23].

In the current study, positive home environmental factors influenced developmental gains over time, despite correction with SEP. Home environment can mediate the relationship between SEP and early childhood development [15, 16] as well as between linear growth and development [23]. The two components of home environment – physical and relational modulate each other, with the sensitive and interactive relational factor overriding the negative effects of a sub-optimal home milieu. The effect of physical overcrowding on childhood development and cognition can be mediated by maternal responsiveness [49].

Maternal factors can influence child development, cognition and behaviour directly and indirectly through better home environmental factors. The effect of maternal depression can even start in the ante-natal period not only through altered placental functions, but also by causing foetal epigenetic changes and stress reactivity [14]. Maternal depression as well as lack of breastfeeding practices can lead to adverse paediatric development [50]. Maternal cognition also affects childhood development and cognition through genetic factors as well as enriching interacting experiences [8, 15, 49].

Iron deficiency in early childhood, when there is an increased iron demand to optimize neuronal maturation, neurotransmitter synthesis, mitochondrial function and other iron dependent enzymes [51], can be detrimental as shown in the current analysis. This early childhood iron deficiency can have persisting effects on cognition in later life as evidenced by another analysis on the same cohort showing cumulative iron deficiency negatively impacting verbal, performance and processing speed domains of cognition at 5 years of age [28]. That assessment had shown that more than 40% of children had iron deficiency at 15 and 24 months of age. Later childhood iron supplementation improves body iron stores, but not the persisting effect of early onset iron deficiency on cognition [51, 52].

There are limitations for the current study including a comparatively small sample size. Though BSID-III is a sensitive and adapted measure, there can be variations (within normal limits) in developmental achievements. However the low-income urban slum setting of the cohort, its high early childhood iron deficiency and a significant decline in developmental quotients over time warrant an assessment as in the current study. Strengths of the study include good follow-up of a longitudinal birth cohort, strong data granularity during early childhood period, standardized developmental, home environment and SEP assessments, blood results from a national reference laboratory and rigorous quality control measures internally and externally.

## Conclusions

This longitudinal birth cohort follow-up study has shown a significant decline in developmental scores in cognition, language, motor and social domains between 6 and 36 months of age in a LMIC urban slum setting. Positive home environmental factors in addition to SEP enhanced developmental outcomes. Both maternal components of cognition and depression showed effects on progress of child development. Linear growth as well as iron deficiency independently influenced development.

Findings of the current study can be relevant for other high risk settings worldwide. The study confirms three large domains associated with childhood development - nurturing environment and SEP under environmental factors, maternal factors of education and depression, and nutritional factors of iron deficiency and appropriate linear growth. Information about appropriate early childhood nutrition and nurturing activities can be provided in both antenatal and immunization clinics to empower mothers. Community level support groups for new mothers can help and support mothers in the post-partum period, while iron supplementation and fortification can optimize early childhood iron deficiency. A triple intervention plan to enhance home environment and nurturance, maternal education and well-being, and early childhood nutrient supplementation might prevent child developmental decline in high risk settings.

## Supplementary Information

**Additional file 1: Supplementary Table 1.** Comparison of baseline characteristics of the birth cohort established in Vellore in 2010.

## Data Availability

MAL-ED data from all sites are deposited in the https://clinepidb.org website, which have public access after appropriate permissions.

## Abbreviations

BSID-III: Bayley Scales of Infant and Toddler Development-III
HOME: Home Observation for the Measurement of the Environment scale
LMIC: Low and middle income countries
MAL-ED: The Etiology, Risk Factors and Interactions of Enteric Infections and Malnutrition and the Consequences for Child Health and Development
SEP: Socio-economic position
SRQ-20: Self Reporting Questionnaire-20
WAMI: Water and sanitation, assets, maternal education and total household income

## References

[1] Grantham-McGregor S, Cheung YB, Cueto S, Glewwe P, Richter L, Strupp B (2007). Developmental potential in the first 5 years for children in developing countries. Lancet.

[2] Black MM, Walker SP, Fernald LCH, Andersen CT, DiGirolamo AM, Lu C, McCoy D, Fink G, Shawar YR, Shiffman J, Devercelli AE, Wodon QT, Vargas-Barón E, Grantham-McGregor S, Lancet Early Childhood Development Series Steering Committee (2017). Early childhood development coming of age: science through the life course. Lancet.

[3] Lu C, Black MM, Richter LM (2016). Risk of poor development in young children in low-income and middle-income countries: an estimation and analysis at the global, regional, and country level. Lancet Glob Health.

[4] Biesalski HK (2016). The 1,000-day window and cognitive development. World Rev Nutr Diet.

[5] Black MM, Pérez-Escamilla R, Rao SF (2015). Integrating nutrition and child development interventions: scientific basis, evidence of impact, and implementation considerations. Adv Nutr.

[6] Georgiadis A, Penny ME (2017). Child undernutrition: opportunities beyond the first 1000 days. Lancet Public Health.

[7] MAL-ED Network Investigators (2018). Early childhood cognitive development is affected by interactions among illness, diet, enteropathogens and the home environment: findings from the MAL-ED birth cohort study. BMJ Glob Health.

[8] McCormick BJJ, Richard SA, Caulfield LE, Pendergast LL, Seidman JC, Koshy B (2019). Early life child micronutrient status, maternal reasoning, and a nurturing household environment have persistent influences on child cognitive development at age 5 years: results from MAL-ED. J Nutr.

[9] Zhu Z, Chang S, Cheng Y, Qi Q, Li S, Elhoumed M, Yan H, Dibley MJ, Fawzi WW, Zeng L, Sudfeld CR (2019). Early life cognitive development trajectories and intelligence quotient in middle childhood and early adolescence in rural western China. Sci Rep.

[10] Prado EL, Dewey KG (2014). Nutrition and brain development in early life. Nutr Rev.

[11] Humphrey JH, Jones AD, Manges A, Mangwadu G, Maluccio JA, Mbuya MN (2015). The Sanitation Hygiene Infant Nutrition Efficacy (SHINE) trial: rationale, design, and methods. Clin Infect Dis.

[12] Ajjampur SSR, Koshy B, Venkataramani M, Sarkar R, Joseph AA, Jacob KS, Ward H, Kang G (2011). Effect of cryptosporidial and giardial diarrhoea on social maturity, intelligence and physical growth in children in a semi-urban slum in South India. Ann Trop Paediatr.

[13] Tarleton JL, Haque R, Mondal D, Shu J, Farr BM, Petri WA (2006). Cognitive effects of diarrhea, malnutrition, and Entamoeba histolytica infection on school age children in Dhaka, Bangladesh. Am J Trop Med Hyg.

[14] Herba CM, Glover V, Ramchandani PG, Rondon MB (2016). Maternal depression and mental health in early childhood: an examination of underlying mechanisms in low-income and middle-income countries. Lancet Psychiat.

[15] Ronfani L, Vecchi Brumatti L, Mariuz M, Tognin V, Bin M, Ferluga V, Knowles A, Montico M, Barbone F (2015). The complex interaction between home environment, socioeconomic status, maternal IQ and early child neurocognitive development: a multivariate analysis of data collected in a newborn cohort study. PLoS One.

[16] Contreras D, González S (2015). Determinants of early child development in Chile: health, cognitive and demographic factors. Int J Educ Dev.

[17] Lynch JL, Gibbs BG (2017). Birth weight and early cognitive skills: can parenting offset the link?. Matern Child Health J.

[18] Nicholson JM, Rempel LA (2004). Australian and New Zealand birth cohort studies: breadth, quality and contributions. J Paediatr Child Health.

[19] Takahashi Y, Okada K, Hoshino T, Anme T (2015). Developmental trajectories of social skills during early childhood and links to parenting practices in a Japanese sample. PLoS One.

[20] Kar BR, Rao SL, Chandramouli BA, Thennarasu K (2011). Growth patterns of neuropsychological functions in Indian children. Front Psychol.

[21] Veena SR, Krishnaveni GV, Wills AK, Kurpad AV, Muthayya S, Hill JC, Karat SC, Nagarajaiah KK, Fall CHD, Srinivasan K (2010). Association of birthweight and head circumference at birth to cognitive performance in 9- to 10-year-old children in South India: prospective birth cohort study. Pediatr Res.

[22] Adair LS, Fall CH, Osmond C, Stein AD, Martorell R, Ramirez-Zea M, Sachdev HS, Dahly DL, Bas I, Norris SA, Micklesfield L, Hallal P, Victora CG, COHORTS group (2013). Associations of linear growth and relative weight gain during early life with adult health and human capital in countries of low and middle income: findings from five birth cohort studies. Lancet.

[23] Black MM, Yimgang DP, Hurley KM, Harding KB, Fernandez-Rao S, Balakrishna N, Radhakrishna KV, Reinhart GA, Nair KM (2019). Mechanisms linking height to early child development among infants and preschoolers in rural India. Dev Sci.

[24] MAL-ED Network Investigators (2014). The MAL-ED study: a multinational and multidisciplinary approach to understand the relationship between enteric pathogens, malnutrition, gut physiology, physical growth, cognitive development, and immune responses in infants and children up to 2 years of age in resource-poor environments. Clin Infect Dis.

[25] John SM, Thomas RJ, Kaki S, Sharma SL, Ramanujam K, Raghava MV, Koshy B, Bose A, Rose A, Rose W, Ramachandran A, Joseph AJ, Babji S, Kang G (2014). Establishment of the MAL-ED birth cohort study site in Vellore, southern India. Clin Infect Dis.

[26] Koshy B, Karthikeyan A, Bose A, Roshan R, Ramanujam K, Mohan VR (2021). Home environment: short-term trends and predictors in early childhood from an Indian community birth cohort. Child Care Health Dev.

[27] Koshy B, Srinivasan M, Murugan TP, Bose A, Christudoss P, Mohan VR, John S, Roshan R, Kang G (2021). Association between head circumference at two years and second and fifth year cognition. BMC Pediatr.

[28] Koshy B, Srinivasan M, Zachariah SM, Karthikeyan AS, Roshan R, Bose A, Mohan VR, John S, Ramanujam K, Muliyil J, Kang G (2020). Body iron and lead status in early childhood and its effects on development and cognition: a longitudinal study from urban Vellore. Public Health Nutr.

[29] Bayley N (2005). Bayley scales of infant and toddler development III.

[30] Murray-Kolb LE, Rasmussen ZA, Scharf RJ, Rasheed MA, Svensen E, Seidman JC, Tofail F, Koshy B, Shrestha R, Maphula A, Vasquez AO, da Costa HP, Yousafzai AK, Oria RB, Roshan R, Bayyo EB, Kosek M, Shrestha S, Schaefer BA, Bessong P, Ahmed T, Lang D, The MAL-ED Network Investigators (2014). The MAL-ED cohort study: methods and lessons learned when assessing early child development and caregiving mediators in infants and young children in 8 low- and middle-income countries. Clin Infect Dis.

[31] Psaki SR, Seidman JC, Miller M, Gottlieb M, Bhutta ZA, Ahmed T (2014). Measuring socioeconomic status in multicountry studies: results from the eight-country MAL-ED study. Popul Health Metr.

[32] Caldwell B, Bradley R (1984). Home observation for the measurement of the environment.

[33] Elardo R, Bradley RH (1981). The home observation for measurement of the environment (HOME) scale: a review of research. Dev Rev.

[34] Black MM, Baqui AH, Zaman K, Ake Persson L, El Arifeen S, Le K (2004). Iron and zinc supplementation promote motor development and exploratory behavior among Bangladeshi infants. Am J Clin Nutr.

[35] Jones PC, Pendergast LL, Schaefer BA, Rasheed M, Svensen E, Scharf R, Shrestha R, Maphula A, Roshan R, Rasmussen Z, Seidman JC, Murray-Kolb LE (2017). Measuring home environments across cultures: invariance of the HOME scale across eight international sites from the MAL-ED study. J Sch Psychol.

[36] Raven J, Raven JC, Court JH (2003). Manual for Raven's progressive matrices and vocabulary scales.

[37] Beusenberg M, Orley JA (1994). Users guide to the self reporting questionnaire (SRQ).

[38] Pendergast LL, Scharf RJ, Rasmussen ZA, Seidman JC, Schaefer BA, Svensen E, Tofail F, Koshy B, Kosek M, Rasheed MA, Roshan R, Maphula A, Shrestha R, Murray-Kolb LE, MAL-ED Network Investigators (2014). Postpartum depressive symptoms across time and place: structural invariance of the self-reporting questionnaire among women from the international, multi-site MAL-ED study. J Affect Disord.

[39] Cook JD, Flowers CH, Skikne BS (2003). The quantitative assessment of body iron. Blood.

[40] Lee J, Choi J-Y (2016). Texas hospitals with higher health information technology expenditures have higher revenue: a longitudinal data analysis using a generalized estimating equation model. BMC Health Serv Res.

[41] Pollitt E, Triana N (1999). Stability, predictive validity, and sensitivity of mental and motor development scales and pre-school cognitive tests among low-income children in developing countries. Food Nutr Bull.

[42] Spittle AJ, Spencer-Smith MM, Eeles AL, Lee KJ, Lorefice LE, Anderson PJ, Doyle LW (2013). Does the Bayley-III motor scale at 2 years predict motor outcome at 4 years in very preterm children?. Dev Med Child Neurol.

[43] Gould JF, Hunt E, Roberts RM, Louise J, Collins CT, Makrides M (2019). Can the Bayley scales of infant development at 18 months predict child behaviour at 7 years?. J Paediatr Child Health.

[44] Koshy B, Mary TTH, Samuel P, Sarkar R, Kendall S, Kang G (2017). Seguin form board as an intelligence tool for young children in an Indian urban slum. Fam Med Commun Health J.

[45] Danaei G, Andrews KG, Sudfeld CR, Fink G, McCoy DC, Peet E (2016). Risk factors for childhood stunting in 137 developing countries: a comparative risk assessment analysis at global, regional, and country levels. PLoS Med.

[46] Sudfeld CR, Charles McCoy D, Danaei G, Fink G, Ezzati M, Andrews KG, Fawzi WW (2015). Linear growth and child development in low- and middle-income countries: a meta-analysis. Pediatrics.

[47] Hamadani JD, Tofail F, Huda SN, Alam DS, Ridout DA, Attanasio O, Grantham-McGregor SM (2014). Cognitive deficit and poverty in the first 5 years of childhood in Bangladesh. Pediatrics.

[48] Hackman DA, Farah MJ, Meaney MJ (2010). Socioeconomic status and the brain: mechanistic insights from human and animal research. Nat Rev Neurosci.

[49] Evans GW, Ricciuti HN, Hope S, Schoon I, Bradley RH, Corwyn RF (2009). Crowding and cognitive development: the mediating role of maternal responsiveness among 36-month-old children. Environ Behav.

[50] Lee KW, Ching SM, Hoo FK, Ramachandran V, Chong SC, Tusimin M, Mohd Nordin N, Devaraj NK, Cheong AT, Chia YC (2020). Neonatal outcomes and its association among gestational diabetes mellitus with and without depression, anxiety and stress symptoms in Malaysia: a cross-sectional study. Midwifery.

[51] Jảuregui-Lobera I (2014). Iron deficiency and cognitive functions. Neuropsychiatr Dis Treat.

[52] Beard JL, Connor JR (2003). Iron status and neural functioning. Ann Rev Nutr.

