# Supplementary material for: Developmental trends in early childhood and their predictors from an Indian birth cohort
Source: BMC Public Health. 2021 Jun 6;21:1083. doi: 10.1186/s12889-021-11147-3 (PMC8180095; doi:10.1186/s12889-021-11147-3)
Supplement: Supplementary file 1 — Additional file 1: Supplementary Table 1. Comparison of baseline characteristics of the birth cohort established in Vellore in 2010. [file 12889_2021_11147_MOESM1_ESM.docx]

**Supplementary table.1**. Comparison of baseline characteristics of the birth cohort established in Vellore in 2010

| Variables | 6 months (n=235) | 15 months  (n=229) | 24 months  (n=228) | 36 months (n=218) |
| --- | --- | --- | --- | --- |
| Sex (%) | | | | |
| Male | 106 (45.11) | 105 (45.85) | 105 (46.05) | 102 (46.79) |
| Female | 129(54.89) | 124 (54.15) | 123 (53.95) | 116 (53.21) |
| Socioeconomic position at 6 months (%) | | | | |
| Low | 71 (30.21) | 70 (30.57) | 69 (30.26) | 66 (30.28) |
| Middle | 80 (34.04) | 78 (34.06) | 78 (34.21) | 74 (33.94) |
| High | 84 (35.74) | 81 (35.37) | 81 (35.53) | 78 (35.78) |
| Maternal depression (SRQ) score at 6 months, mean (SD) | 4.53 (3.74) | 4.49 (3.77) | 4.51 (3.76) | 4.52 (3.77) |
| Maternal cognition raw score, mean (SD) | 43.90 (10.49) | 43.87 (10.50) | 43.86 (10.52) | 43.67 (10.57) |
| Stunting at 6 months* (%) |  |  |  |  |
| Yes | 46 (19.57) | 46 (20.26) | 46 (20.18) | 43 (19.72) |
| No | 187 (79.57) | 181 (79.74) | 180 (78.95) | 173 (79.36) |
| Underweight at 6 months*(%) |  |  |  |  |
| Yes | 54 (22.98) | 53 (23.35) | 53 (23.25) | 51 (23.39) |
| No | 179 (76.17) | 174 (76.65) | 173 (75.88) | 165 (75.69) |
| Body iron at 7 months, mean (SD) | 5.42 (5.06) | 5.40 (5.05) | 5.41 (5.06) | 5.47 (5.14) |

*Two children had a missing data on stunting and overweight status measured at 6 months.
